# Bacteria- and IMD Pathway-Independent Immune Defenses against *Plasmodium falciparum* in *Anopheles gambiae*

**DOI:** 10.1371/journal.pone.0072130

**Published:** 2013-09-03

**Authors:** Benjamin J. Blumberg, Stefanie Trop, Suchismita Das, George Dimopoulos

**Affiliations:** W. Harry Feinstone Department of Molecular Microbiology and Immunology, Bloomberg School of Public Health, Johns Hopkins University, Baltimore, Maryland, United States of America; Centro de Pesquisas René Rachou, Brazil.

## Abstract

The mosquito *Anopheles gambiae* uses its innate immune system to control bacterial and *Plasmodium* infection of its midgut tissue. The activation of potent IMD pathway-mediated anti-*Plasmodium falciparum* defenses is dependent on the presence of the midgut microbiota, which activate this defense system upon parasite infection through a peptidoglycan recognition protein, PGRPLC. We employed transcriptomic and reverse genetic analyses to compare the *P. falciparum* infection-responsive transcriptomes of septic and aseptic mosquitoes and to determine whether bacteria-independent anti-*Plasmodium* defenses exist. Antibiotic treated aseptic mosquitoes mounted molecular immune responses representing a variety of immune functions upon *P. falciparum* infection. Among other immune factors, our analysis uncovered a serine protease inhibitor (*SRPN7*) and Clip-domain serine protease (*CLIPC2*) that were transcriptionally induced in the midgut upon *P. falciparum* infection, independent of bacteria. We also showed that *SRPN7* negatively and *CLIPC2* positively regulate the anti-*Plasmodium* defense, independently of the midgut-associated bacteria. Co-silencing assays suggested that these two genes may function together in a signaling cascade. Neither gene was regulated, nor modulated, by infection with the rodent malaria parasite *Plasmodium berghei*, suggesting that *SRPN7* and *CLIPC2* are components of a defense system with preferential activity towards *P. falciparum*. Further analysis using RNA interference determined that these genes do not regulate the anti-*Plasmodium* defense mediated by the IMD pathway, and both factors act as agonists of the endogenous midgut microbiota, further demonstrating the lack of functional relatedness between these genes and the bacteria-dependent activation of the IMD pathway. This is the first study confirming the existence of a bacteria-independent, anti-*P. falciparum* defense. Further exploration of this anti-*Plasmodium* defense will help clarify determinants of immune specificity in the mosquito, and expose potential gene and/or protein targets for malaria intervention strategies based on targeting the parasite in the mosquito vector.

## Introduction

Human malaria continues to be a scourge of mankind, responsible for approximately over a million deaths annually on average [1]. *Plasmodium falciparum,* the most dangerous malaria parasite, is responsible for the majority of deaths worldwide and is transmitted by the mosquito *Anopheles gambiae* as well as other Anopheline species.

Like other insects, *A. gambiae* relies on its innate immune response to defend against infections with pathogens, including *Plasmodium* [2], [3]. Upon activation of pattern recognition receptors that can bind to microbial pathogen associated molecular patterns, two main signaling pathways, the TOLL and immune deficiency (IMD) pathways, launch effective anti-pathogen immune responses through NF-kappaB transcription factors that transcribe effector genes [4]. The link between pattern recognition receptor and immune pathway activation can either be direct, as in the case of the IMD pathway activation by PGRPLC, or can be indirectly mediated through serine protease cascades that are controlled by serpins, as in the case of TOLL pathway activation. The serine protease cascades also play other roles in immune responses, providing signal amplification that leads to the activation of anti-pathogen effector mechanisms such as melanotic encapsulation [4].

While the TOLL pathway has been shown to suppress infection with the rodent malaria parasite *P. berghei*, the IMD pathway is associated with anti-*P. falciparum* defense and is mediated through the activation of anti-*Plasmodium* effectors such as the fibrinogen-like immunolectin 9 (FBN9), leucine-rich repeat domain containing protein 7 (LRRD7), thioester containing protein 1 (TEP1), and other defense proteins [5]–[10]. Furthermore, we have shown that the IMD pathway-regulated transcription factor Rel2 is controlling the mosquito midgut microbiota while the Toll pathway and its transcription factor Rel1 does not [8], [11]. Finally, while others have shown that the anti-*Plasmodium* IMD pathway is activated by bacteria we have shown that this pathway's (if activated through RNAi of IMD pathway negative regulator Caspar) anti-*Plasmodium* activity is independent on the presence of bacteria [12]. The two pathways are also essential to the defense against fungi and bacteria [4].

Recent studies have shown a dependence on bacteria-mediated activation of the IMD pathway to launch an effective anti-*Plasmodium* immune response in the mosquito gut tissue, which harbors a variety of mostly Gram-negative bacteria [13]. The IMD pathway contributes to microbial homeostasis in this tissue by maintaining a continuous basal level activity, which is stimulated by the midgut microbiota [11]. PGRPLC, a peptidoglycan recognition protein isoform previously identified as a receptor of the IMD pathway in *Drosophila*, has been shown to play a role in this basal level immune activation by sensing bacteria in the gut tissue [11], [13].

Interestingly, PGRPLC has also been shown to be essential for triggering an anti-*P. falciparum* response through the IMD pathway, but only when bacteria are present in the gut tissue [13]. RNAi-mediated depletion of PGRPLC from antibiotic-treated *A. gambiae,* which have a greatly reduced microbial flora, does not influence the mosquito's susceptibility to the parasite, as it does in non-antibiotic-treated septic mosquitoes, suggesting that PGRPLC–mediated activation of the IMD pathway's anti-*Plasmodium* defense depends on the presence of midgut bacteria [11], [13]. We and others have previously shown that the anti-*Plasmodium* effectors FBN9, LRRD7, and TEP1 are also involved in controlling bacterial proliferation in the midgut tissue, corroborating the intimate relationship between anti-bacterial and anti-*Plasmodium* defenses [5], [11]. Here we wanted to investigate whether *P. falciparum* ookinete infection of the mosquito midgut activates anti-parasitic immune responses in a bacteria-independent manner. Arrighi *et al*. previously investigated the role of *Plasmodium* glycosylphosphatidylinositol (GPI) anchors in the induction of an immune response, and although their study documented the induction of some immune genes, the potential anti-*Plasmodium* action and possible dependence and relationship of these genes to the microbiota were not investigated [14].

Since the transcriptome of an organism, tissue, or cell type represents a reflection of a physiological state such as immune response, we used whole-genome microarray analysis to investigate the *P. falciparum* infection-responsive transcriptome in septic and aseptic mosquitoes in order to identify and characterize bacteria-independent immune response signatures and factors. Our analysis revealed a variety of putative immune genes that are regulated upon *Plasmodium* infection in the absence of midgut microbiota, and we specifically focused on a clip-domain serine protease (*CLIPC2*) and a serine protease inhibitor (*SRPN7*), showing that these genes modulate the intensity of the *P. falciparum* infection in the absence of bacteria. *CLIPC2* also controlled systemic bacterial infection and both genes modulated the proliferation of the midgut microflora, indicating their functional versatility. Interestingly, our study suggests that CLIPC2 and SRPN7 may be part of the same protease signaling cascade and that their anti-*P. falciparum* function is independent of the IMD pathway, since these factors were not regulated by and did not regulate this pathway. Our study points to the existence of bacteria-independent anti-*Plasmodium* defenses, possibly relating to as-yet unknown immune pathways and mechanisms.

## Materials and Methods

### Ethics Statement

This study was carried out in strict accordance with the recommendations in the Guide for the Care and Use of Laboratory Animals of the National Institutes of Health. The protocol was approved by the Animal Care and Use Committee of the Johns Hopkins University (ACUC: MO11H184). Commercial anonymous human blood was obtained from Interstate Bloodbank and used for parasite cultures and mosquito feeding and informed consent was therefore not applicable. The Johns Hopkins School of Public Health Ethics Committee has approved this protocol.

### Mosquito Rearing, RNA Isolation, and cDNA Synthesis

*A. gambiae* Keele strain mosquitoes [15] were maintained on a 10% sucrose solution with 12-h light/dark cycles at 27°C and 80% humidity [16]. At specific time points, mosquitoes were anesthetized on ice, and either whole mosquitoes or specific tissues were dissected and collected. RNA was extracted from tissues using the RNeasy kit (Qiagen), and RNA yields were quantified using the Nanodrop 2000 (ThermoFisher) following treatment with DNase (Ambion) cDNA was synthesized from extracted RNA using the Oligo-DT primer and M-MLV Reverse Transcriptase (Promega).

### Primer Design and qRT-PCR

The Primer 3 Program (http://frodo.wi.mit.edu) was used to design all primers, except for the bacterial *16s* primers [17]. Real-time quantitative PCR (qRT-PCR) to assess transcript abundance and silencing efficiency was performed as described in [5]. Transcript abundance was quantified with Sybr Green PCR Master Mix (Applied Biosystems) using the ABI StepOnePlus Real-Time PCR System and ABI StepOne Software. PCR reactions were performed in duplicate, and melting curve analysis was used to analyze primer specificity. Transcript abundance of target genes were first normalized to the within sample transcript abundance of the mosquito ribosomal *S7* gene, and fold changes between samples were determined using the ΔΔct method.

### RNAi Gene Silencing

The HiScribe T7 *in vitro* Transcription Kit (New England Biolabs) was used to generate double-stranded RNA (dsRNA) from PCR-amplified gene oligos. Gene silencing was performed essentially as described in [5], [18]. In brief, 3- to-4-day-old female *A. gambiae* were cold-anesthetized, and cohorts were injected with control *GFP* dsRNA or experimental gene-specific dsRNA at a concentration of 3 µg/µL (207 ng dsRNA per mosquito). Pools of 15 mosquitoes were collected 1–4 days post-dsRNA injection, and silencing efficiency was assessed by qRT-PCR.

### Quantification of Endogenous Mosquito Midgut Bacteria

Colony forming units (CFU) from mosquito midguts were quantified in control untreated, antibiotic-treated, and gene-silenced mosquitoes as described [11], [19]. For the gene silencing experiment, 3–4 day old female *A. gambiae* mosquitoes were injected with dsRNA as described in the section “RNAi Gene Silencing.” 3 days post-injection of dsRNA, mosquito midguts were dissected and processed as described below. At the indicated time points, female mosquitoes were collected, surface-sterilized in ethanol, and washed with 1x PBS, and their midguts were dissected in sterilized 1x PBS. Collected midguts were homogenized, and serial dilutions of homogenate were plated on LB agar plates. After incubation for 2–3 days at 27°C under aerobic or anaerobic conditions, the CFUs per plate were counted, and a titer of CFU/midgut was calculated.

### Antibiotic Treatment

For antibiotic treatment, adult female mosquitoes were collected post-eclosion and given a sterile 10% sucrose solution containing 75 μg/mL gentamicin sulfate (Quality Biological) and 100 units (μg)/mL of penicillin-streptomycin (Invitrogen). Treatment was carried out for at least 3 days, and antibiotic-containing sucrose was changed daily to ensure adequate elimination of bacteria. To validate the efficiency of antibiotic treatment, midguts from control untreated and experimental antibiotic treated mosquitoes were subjected to culture-dependent CFU and culture-independent enumeration assays. The culture-dependent CFU assay tested sugar-fed, antibiotic-treated sugar-fed, blood-fed, and antibiotic-treated blood-fed mosquito midguts under aerobic or anaerobic conditions: Individual midguts were collected from sugar-fed and blood-fed (24 h post-blood feeding) adult females essentially as described above. Individual midgut samples were homogenized in 1x PBS, and serial dilutions of the homogenate were spread on LB agar plates cultured under aerobic or anaerobic conditions. Anaerobic conditions were achieved using the BD GasPak EZ Anaerobe Container System (BD). The culture-independent assay involved qRT-PCR of the bacterial 16s ribosomal gene from the two sugar-fed and two blood-fed groups listed above.

### *Plasmodium* Challenge

*P. falciparum* and *P. berghei* challenges were accomplished following a standard protocol [5]. For *P. falciparum* infection: Three days post-dsRNA injection, mosquitoes fed on NF54W strain gametocytes in human blood through a membrane feeder at 37°C. Unfed mosquitoes were removed within the first day post-infection, and engorged mosquitoes were maintained at 27°C for up to 8 days. For *P. berghei* infection: Three days post-dsRNA injection, mosquitoes were allowed to feed on Swiss Webster mice infected with the WT Anka 2.34 strain of the parasite. Unfed mosquitoes were removed within the first day post-infection, and engorged mosquitoes were maintained at 19°C for 14 days. *P. falciparum-* and *P. berghei*-infected mosquito midguts were dissected and stained with 0.1% mercurochrome, and oocyst numbers were counted using a light microscope (Olympus).

### Microarray Hybridization and Data Analysis

All probe sequences, probe preparation, microarray construction, and microarray hybridizations were performed essentially as described in [5]. Control (Cy3-labeled) and experimental (Cy5-labeled) cRNA probes were generated from 2–3 μg of RNA according to the manufacturer's instructions (Agilent Technologies Low RNA Input Linear Amplification Kit). Probe hybridization to the microarray slides was accomplished using 2 μg of cRNA, and microarray slides were washed and dried 16 h post-hybridization. Slides were scanned using an Axon GenePix 4200AL scanner at 10-μm pixel size (Axon Instruments, Union City, California, USA); 60% laser power was used, and the photomultiplier tube (PMT) voltage was adjusted to maximize the dynamic range and minimize pixel saturation. GenePix software was used to analyze the scanned images. Cy5 and Cy3 values were processed and subjected to statistical analysis using the TIGR, MIDAS, and TMEV software packages [20]. The minimum signal intensity accepted was 100 fluorescent units, and a signal-to-background cutoff ratio of 2.0 was used. Three biological replicates and a pseudo replicate were performed for each group. Median fluorescent values for good spots were normalized by the LOWESS normalization method [21]. Statistical analysis of Cy5/Cy3 ratios was performed using a *t*-test with significance at p < 0.05, and the cutoff value for significant gene regulation was 0.75 on a log2 scale [21]. The microarray data was assembled in the Minimum Information About a Microarray Experiment (MIAME)-compliant format and is available in the public Gene Expression Omnibus (GEO) database under accession GSE49690.

### Bacterial Challenge

Mosquitoes were injected with control ds*GFP* or experimental dsRNA constructs. Three days post-injection, mosquitoes were cold-anesthetized and injected with 69 nL of either Gram-positive *Staphylococcus aureus* or Gram-negative *Escherichia coli*, at the optical density mentioned at the end of this section. Glycerol stocks of both species of bacteria were used to inoculate LB broth cultures, which were grown in a shaker overnight at 37°C for approximately 18 h. The cultures were centrifuged to a pellet that was then washed with 1x PBS three times. A biophotometer (Eppendorf) was used to measure optical density (OD600), and samples were diluted with 1x PBS to the appropriate absorbance prior to injection (OD600; *S. aureus*  = 0.35, *E. coli* = 3.0).

### Statistical Analysis

The Graphpad Prism 5 (Graphpad Prism®) software package was used to perform statistical analyses. The particular test used is indicated in the captions of each respective figure.

## Results and Discussion

### Bacteria-dependent and -independent mosquito transcriptome responses to *P. falciparum* infection

**Figure 1:**
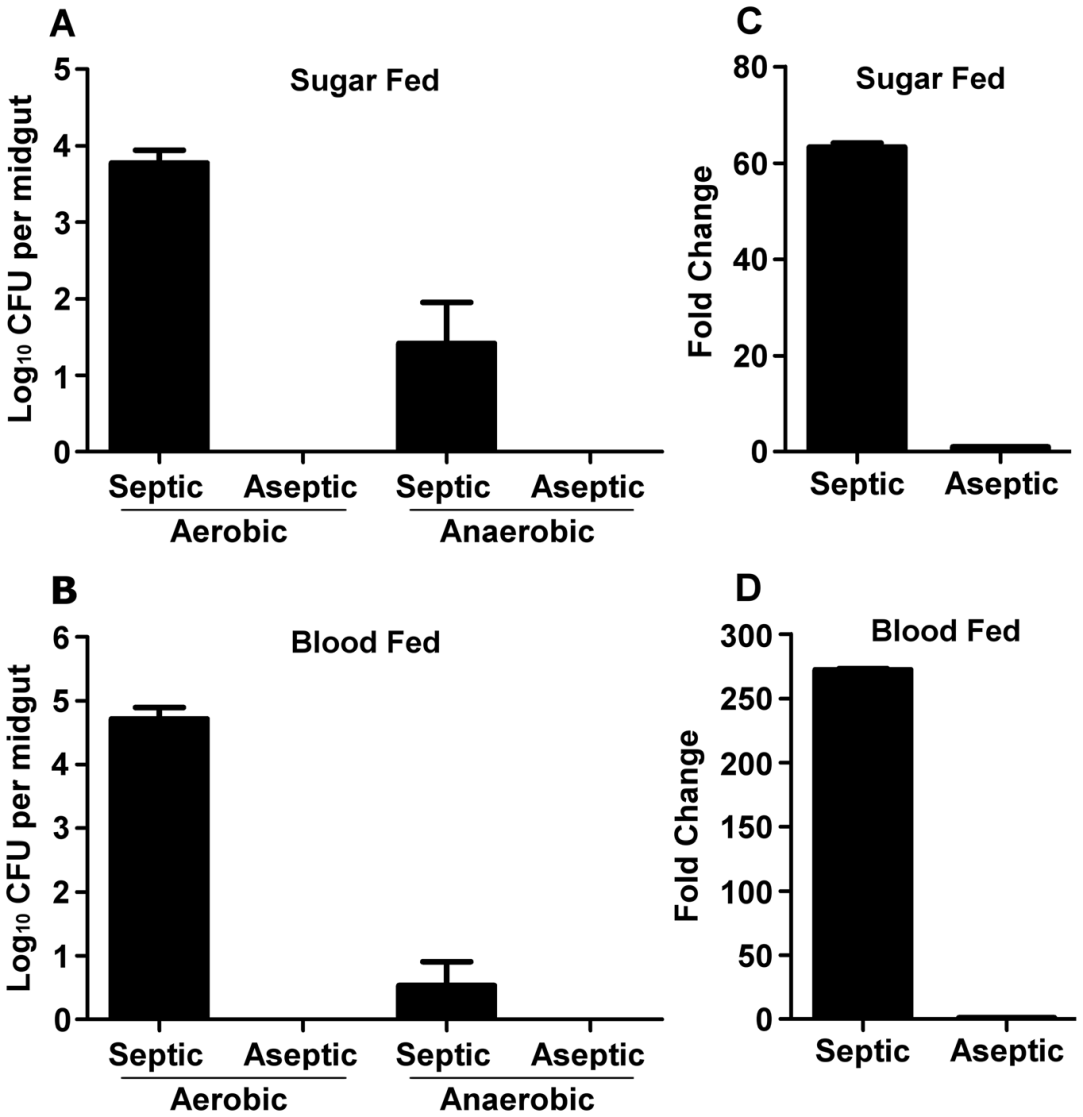
Removal of bacteria from the midgut by antibiotic treatment of adult female mosquitoes. Culture-dependent methods of bacterial cultivation (aerobic vs. anaerobic conditions) were unsuccessful at growing any bacteria from the midguts of aseptic (antibiotic-treated) mosquitoes after feeding on either (A) sugar or (B) 24 h post-blood meal. In contrast, bacteria from the midguts of septic (untreated) mosquitoes fed on (A) sugar or (B) 24 h post-blood meal could be cultured under aerobic and anaerobic conditions. (C) and (D) Culture-independent analysis of bacterial 16s rRNA by qRT-PCR measured almost no 16s rRNA in aseptic mosquitoes (sugar or blood-fed). For (A) and (B), colony forming units (CFU) from three biological replicates were pooled. For (C) and (D), 10 midguts from each treatment were assessed individually by qRT-PCR, and the relative amount of 16s rRNA from aseptic midguts (sugar or blood-fed) was compared to that of the septic (sugar or blood-fed) midgut groups, respectively. Black bars represent the mean CFU or mean -fold change, and error bars represent the standard error of the mean.

To examine the impact of *P. falciparum* infection on the mosquito midgut and carcass transcriptomes in the presence or absence of midgut bacteria, we used *A. gambiae* whole genome microarrays to compare the mRNA abundance of *P. falciparum*-infected and -naïve mosquitoes of antibiotic- and non-antibiotic treated cohorts. Depletion of the great majority of midgut bacteria was achieved by treating mosquitoes with a broad-spectrum antibiotic cocktail containing 75 ug/ml gentamycin, 100 units/ml penicillin and 100 ug/ml streptomycin for 4 days through their sugar meal, prior to feeding on *P. falciparum* gametocytes. To assess the efficacy of the antibiotic treatment in the removal of the midgut microbiota, we assayed colony forming unit (CFU) growth on LB agar of both the aerobic and anaerobic bacteria present in sugar-fed and 24-h blood-fed mosquito midguts (Figure 1A, B). Although culturing bacterial isolates exclusively on LB agar may limit the ability to capture the entire spectrum of bacterial species present in the mosquito midgut, we have observed near identical growth of the same bacteria on a variety of mediums (LB, Yeast extract-peptone dextrose, blood agar), (Dimopoulos lab, unpublished data). Our assays showed that no CFU could be detected in antibiotic-treated mosquitoes. Since some midgut bacteria may be unculturable we alsowe determined the relative microbial load of these samples using qRT-PCR with universal primers amplifying the bacterial 16s ribosomal RNA (16s rRNA), (Table S1). The 16s rRNA was amplified 63-fold higher in septic sugar-fed and 272-fold higher in septic blood-fed midguts when normalized to 16s rRNA from aseptic sugar-fed and aseptic blood-fed midguts, respectively (Figure 1C, D). This PCR-amplified bacterial nucleic acid could represent the remains of dead bacteria that existed in the midguts prior to the antibiotic treatment, and the large difference in 16s rRNA abundance between septic and aseptic midguts suggests near complete removal of the microbiota by antibiotic treatment. It is possible that RNA was amplified from a small number of bacteria unaffected by antibiotic treatment. Nevertheless, we have previously demonstrated that the removal of bacteria through antibiotic treatment is efficient to impact on the *Plasmodium* infection phenotype [11], and for practical purposes we consider our antibiotic-treated mosquitoes aseptic when compared non-antibiotic treated septic mosquitoes.

**Figure 2:**
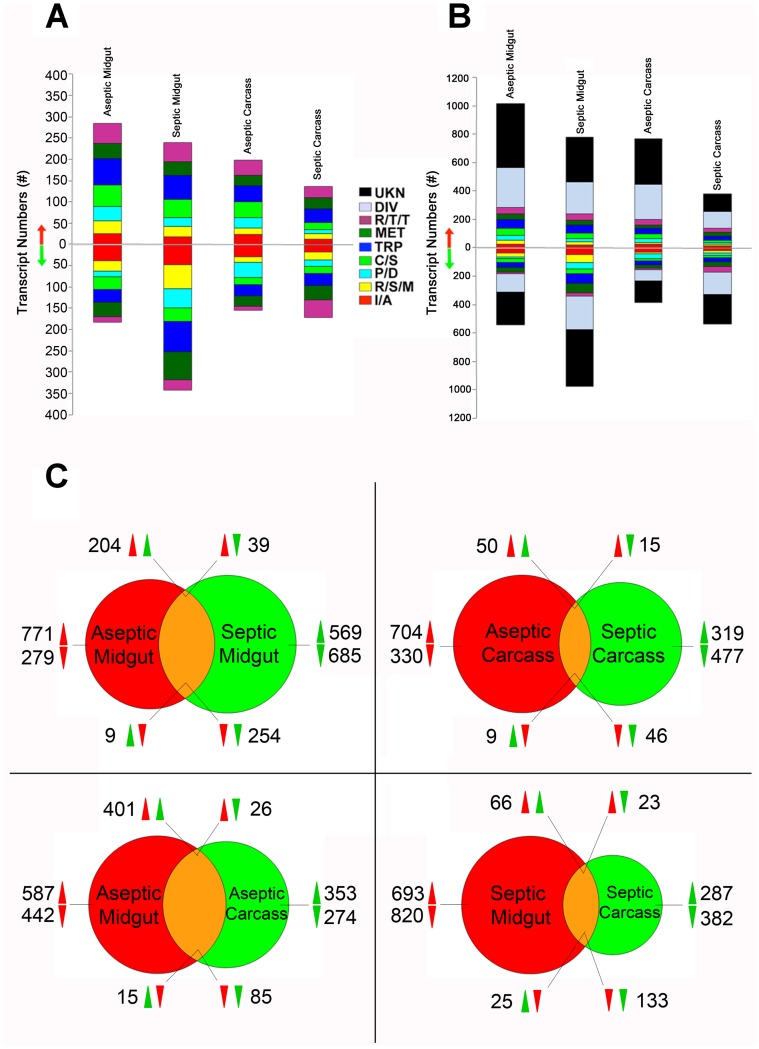
Global gene regulation of mosquitoes at 24 h post-*P. falciparum* infection under septic and aseptic conditions. (A) Numbers of up- or down-regulated genes in distinct functional groups according to tissue (midgut/carcass) and treatment (septic/aseptic) at 24 h post-*P. falciparum* infection (not including DIV/UKN). (B) Same as in (A) but including DIV/UKN. (C) Venn diagrams comparing the total numbers of regulated genes between tissues and treatments. Red arrows correspond to the tissues/treatments in the left circles, and green arrows correspond to the tissues/treatments in the right circles. The arrow direction indicates up- or down-regulation. I/A: putative immunity and apoptosis; R/S/M: oxidoreductive, stress-related and mitochondrial; C/S: cytoskeletal, structural; MET: metabolism; R/T/T: replication, transcription, translation; P/D: proteolysis, digestion; TRP: transport; DIV: diverse; UKN: unknown functions.

We then compared the genome-wide transcript abundance between infected and non-infected mosquitoes of septic and aseptic cohorts at 24 h post-ingestion of a *P. falciparum* gametocyte culture or naïve blood; a time period when ookinetes invade the midgut epithelium and the microbial flora has expanded by a 10- to 20-fold in the nutrient-rich blood (Figure 2) [22], [23]. Regulation was determined by assessing Log2 ratio values of transcript abundance that were above our cutoff for significance of 0.75, or below −0.75. *P. falciparum* infection induced changes in the abundance of as many as 2,183 and 2,429 transcripts in whole mosquitoes belonging to a variety of functional groups in aseptic and septic mosquitoes, respectively, representing approximately 16% and 18% of the *A. gambiae* transcriptome (Figure 2A, B). The abundance of 1,556 transcripts was regulated in the aseptic midguts, as compared to 1,760 in the septic midguts, and 1,154 and 916 transcripts displayed changes in the aseptic and septic carcasses, respectively, upon *P. falciparum* infection (Figure 2C). The abundance of 458 transcripts changed (204 induced and 254 repressed) in the same direction in both aseptic and septic midguts, suggesting that *P. falciparum* infection, and not the presence or absence of the midgut bacteria, was likely to account for this response (Figure 2C). In comparison, the transcript abundance of only 96 genes was similarly regulated (50 induced and 46 repressed) in both the aseptic and septic carcasses, suggesting that *Plasmodium* ookinete invasion and traversal of the midgut has a greater impact on this tissue than on the rest of the mosquito (Figure 2C). The abundance of only 48 transcripts displayed an opposite pattern of change between the aseptic and septic midguts (39 induced in aseptic midguts, 9 repressed in aseptic midguts), suggesting that the presence or absence of midgut bacteria influences the expression of these particular genes after *Plasmodium* infection (Figure 2C). In addition, 46 transcripts with predicted immune functions were uniquely regulated in the septic midgut (11 induced and 35 repressed), suggesting that tripartite interactions between *Plasmodium* parasites, midgut bacteria, and the midgut epithelium affect the expression of this set of genes (Table S2). When we compared transcript abundance between the midgut and carcass within the aseptic group (Figure 2C), we observed 486 regulated genes (401 induced and 85 repressed) shared between these tissue compartments. This is over double the number of regulated transcripts (66 induced and 133 repressed) that were shared between the midgut and carcass within the septic group. This observation suggests that the presence of midgut bacteria accounts for larger differences between the midgut and carcass transcriptomes, whereas in the absence of midgut bacteria there is more similarity in the transcriptome between the two tissue compartments.

Transcripts of a suppressor of cytokine signaling (*SOCS*, AGAP001623, Log2 = 0.97) were upregulated in the *P. falciparum* infected septic midguts, as was a *SOCS* negative regulator of the *A. gambiae* JAK-STAT pathway that had been previously implicated as a host factor in *Plasmodium* infection [24]. The transcripts of the secreted modular serine protease 22D (*SCRASP1*, AGAP005625, Log2 = 0.88) were also upregulated in the septic midguts. SCRASP1 has a chitin-binding domain that has been hypothesized to sense chitin in response to injury and to transduce signals via the serine protease domain, even though the signaling pathway to which *SCRASP1* belongs has remained elusive [25], [26]. Also upregulated in the septic parasite-infected midguts were transcripts of spaetzle-like cytokine 2 (*SPZ2,* AGAP006483, Log2 = 0.75), which may be involved in TOLL pathway activation [27]. Transcripts of the thioester-containing protein 1 (*TEP1*, AGAP010815, Log2 = 0.72), an IMD-pathway associated effector molecule with strong anti-*Plasmodium* activity, was close to being significantly regulated in accordance with previous observations of *P. falciparum* infection in the septic gut [5], [6].

Ultimately, we were interested in identifying the genes involved in bacteria-independent anti-*Plasmodium* responses, and therefore we focused on transcripts displaying increased abundance in the parasite-infected aseptic midguts, placing a particular emphasis on those with predicted immune functions. Of the 783 transcripts specifically enriched in the aseptic midguts, 17 had predicted functions in immunity, whereas the majority of genes of this group belonged to other diverse or unknown functional groups. Two genes that displayed changes in their transcript abundance in *P. falciparum*-infected aseptic midguts, *LRRD1* (AGAP000360, Log2 = 1.14) and *LRRD18* (AGAP000054, Log2 = 1.3), belong to the leucine rich repeat domains (*LRRD*) gene family, which also contains members with a putative function in pattern recognition and to play key roles in anti-*Plasmodium* functions [5], [28], [29]. Fibrinogen-related proteins have been implicated in the pattern recognition processes of human and rodent malaria parasites [30], and two FBN genes (*FBN34* AGAP001554, Log2 = 1.04) and a novel gene, (XM_001231172, AGAP010772, Log2  = 1.86) encoding such putative immune factors were upregulated in the aseptic midguts by parasite infection. Another upregulated gene, *SCRB5* (AGAP002738, Log2 = 3.17), belongs to a class of scavenger receptors with diverse roles in pattern recognition, phagocytosis, and *Plasmodium* infection [31]–[33]. A non-alternatively spliced region of the *AGDSCAM* gene (AGAP007092, Log2 = 1.49) was also upregulated. Transcripts of this gene, in theory, can produce over 31,000 splice forms through alternative splicing, and *AGDSCAM* already has a recognized role in pattern recognition and immunity to *Plasmodium* infection [19]. Also upregulated in the aseptic midguts were a number of serine proteases and serine protease inhibitors. Studies have previously described roles for these gene families in melanization, immune pathway activation, and anti-parasitic activity [34]–[36].

Because of the central role of serine protease cascades in regulating insect immune defenses [37], [38], we focused the remainder of our analysis on a clip-domain serine protease C2 (*CLIPC2*, AGAP004317, Log2 = 0.96) and a serine protease inhibitor 7 (*SRPN7*, AGAP007693, Log2 = 4.16) that were specifically upregulated in the parasite-infected, aseptic mosquito midgut. Their regulation by *P. falciparum* infection in the absence of the midgut microbiota suggested that they were likely to be involved in regulating bacteria-independent anti-*Plasmodium* defenses.

Serpins represent a large family of negative regulators of proteolytic cascades that play a critical roles in a variety of processes both vertebrates and invertebrates [39]. In humans, serpins regulate finely tuned processes such as fibrinolytic cascades, clotting, and inflammatory reactions [40]. In arthropods, serpins have been shown to regulate components of the prophenoloxidase (PPO) pathway, which is responsible for the melanization of pathogens, as well as to inhibit processes upstream of the seminal TOLL pathway, which functions in both development and innate immunity [36], [38], [41]–[43]. *A. gambiae SRPN7* has 1:1 orthologs in both the yellow fever mosquito *Aedes aegypti* and the Southern house mosquito *Culex quinquefasciatus*, suggesting that the gene has a conserved, mosquito-specific function. Clip-Domain serine proteases also belong to a large gene family, but unlike the serine protease inhibitors (serpins), they are only found in arthropods [44], [45]. Functional studies have demonstrated a role for Clip proteases in the activation of prophenoloxidases (PPOs), which mediate melanization defenses as well as the TOLL pathway [38], [46]–[49]. In mosquitoes, there are five sub-families of Clip proteases (A, B, C, D, and E), and studies on subfamily A and B members have shown that some of these genes regulate the PPO pathway [38], [50]–[52]. Although little is known about the role of subfamily C members in mosquitoes, it is worth noting that in *Drosophila* subfamily C members include SNAKE and PERSEPHONE, which are involved in TOLL pathway activation in in the context of development and immunity, respectively [53], [54]. The catalytic triad (His, Asp, Ser) is present in this clip protease indicating likely enzymatic activity similar to what was observed in another clip serine protease [55]. Like *SRPN7*, *CLIPC2* has 1:1 orthologs in both *C. quinquefasciatus* and *A. aegypti,* again suggesting a mosquito-specific gene function.

### *SRPN7* and *CLIPC2* infection-responsiveness

**Figure 3:**
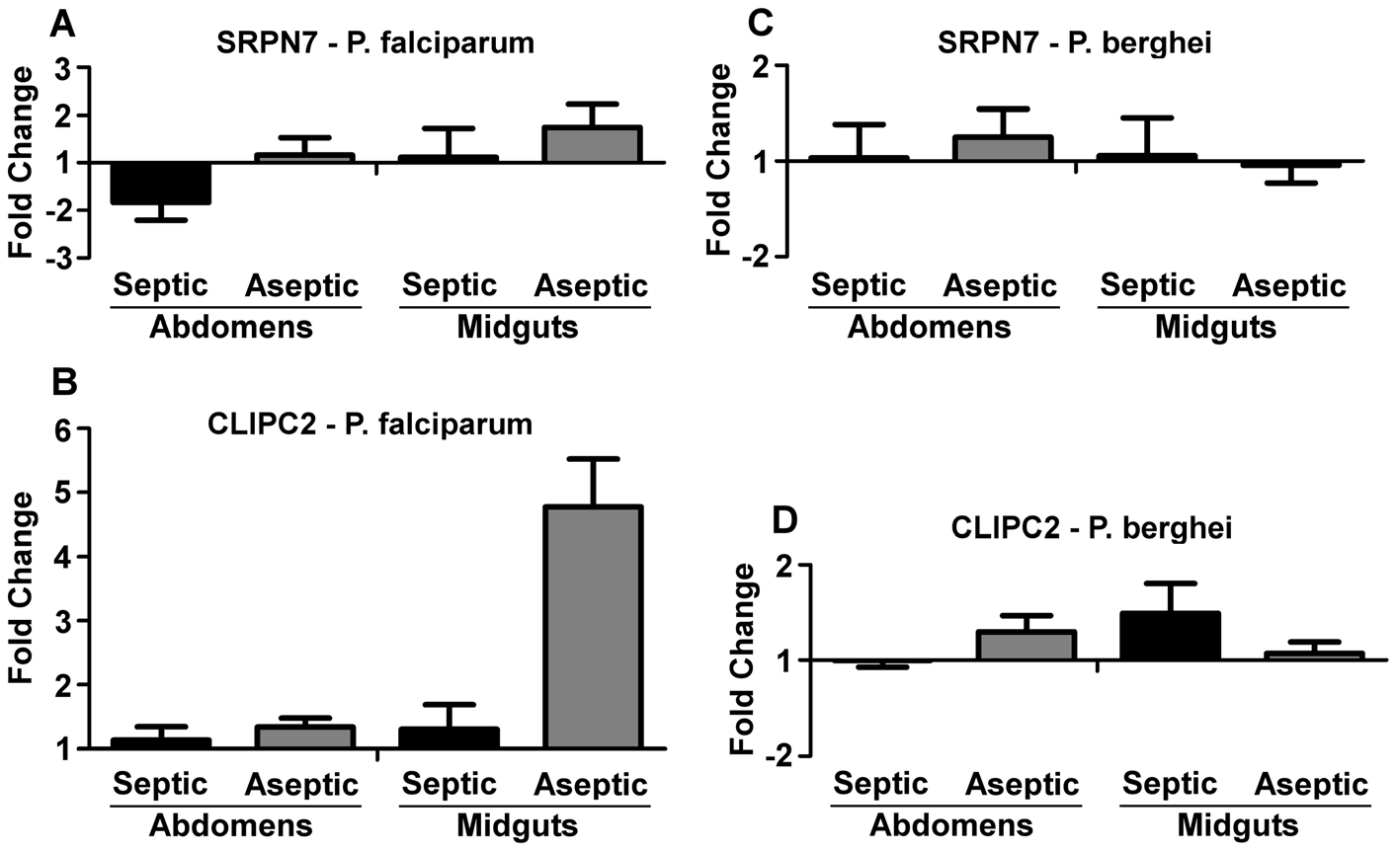
Tissue-specific expression of *SRPN7* and *CLIPC2* after *Plasmodium* infection. Fold change in transcript abundance of (A) *SRPN7* and (B) *CLIPC2* at 24 h post-*P. falciparum* infection. (C) Fold change in expression of *SRPN7* and (D) *CLIPC2* at 24 h post-*P. berghei* infection. Bars represent the mean -fold change in transcript abundance of *SRPN7* and *CLIPC2* between tissues (Midgut/Abdomen) and treatments (Septic/Aseptic) when compared to naïve blood-fed controls of the same tissue/treatment. Data are from three independent biological replicates, and error bars represent the standard error of the mean. Statistical analysis performed by Mann-Whitney test comparing the dCT values of infected to uninfected samples of the same tissue/treatment type resulted in no significant difference between any of the comparisons. There was also no significant difference between tissues when comparing transcript abundance of aseptic to septic samples of the same tissue compartment. These data were processed according to Livak and Schmittgen 2001 [64].

Quantitative real-time PCR (qRT-PCR) assays were used to confirm the up-regulation of *SRPN7* and *CLIPC2* in aseptic *P. falciparum*-infected mosquitoes (Table S1). The infection-responsive increase in *SRPN7* transcript abundance was greatest in the aseptic midgut, although it was modest when compared to that of mosquitoes fed on naïve blood (Figure 3A). Since *SRPN7* transcripts were previously detected at low levels in adult mosquitoes [56], the increase in transcript abundance upon *Plasmodium*-infection of the aseptic midgut is intriguing. *SRPN7* transcripts have previously been reported to be upregulated in the midguts of mosquitoes fed on a blood meal mixed with Gram-positive and Gram-negative bacteria [11]. Analysis of *CLIPC2* has shown nearly a 5-fold increase in transcript abundance after *P. falciparum* infection of aseptic mosquito guts at 24 h after feeding on a gametocyte culture, when compared to mosquitoes fed on naive blood (Figure 3B). Our earlier studies on the IMD pathway-regulated mosquito transcriptome have suggested that *SRPN7* and *CLIPC2* are not regulated by the IMD pathway [6]. It is possible that differences in *P. falciparum* infection intensity and/or prevalence between septic and aseptic mosquitoes could have influenced the transcript abundance of these genes, but it is technically challenging to normalize the amount of pre-invasive parasites between antibiotic-treated and untreated cohorts. Nevertheless, our data prove bacteria-independent induction of *SRPN7* and *CLIPC2* upon *P. falciparum* infection. Overall, the pattern of gene transcript regulation detected by qRT-PCR supported our microarray-based studies, confirming that these genes are not influenced by parasitic infection in septic mosquito guts or carcasses and suggesting the occurrence of a bacteria-independent induction by parasite infection.

Interestingly, the transcript abundance of these genes was not modulated by infection of either septic or aseptic mosquitoes with the rodent malaria parasite *P. berghei*, suggesting a possible functional role in parasite species-specific immune defenses (Figure 3C, D). *P. falciparum* and *P. berghei* exhibit stark differences in their development within the mosquito that may explain the *P. falciparum*-specific induction observed in this study [57]. The lack of transcript abundance modulation in parasite-infected septic mosquitoes is interesting and may indicate that these genes are also regulated by bacteria in a way that counteracts or masks induction by *P. falciparum* when compared to the non-infected mosquitoes. Interestingly, *CLIPC2* was previously reported to be induced in the midguts of mosquitoes fed heat-killed *E. coli* [58], suggesting that *CLIPC2* is upregulated in both a bacteria-dependent and bacteria-independent but parasite-dependent manner. The differential regulation of CLIPC2 by *P. berghei* and *P. falciparum* infection of aseptic mosquitoes does support parasite-mediated regulation rather than an exclusive dependence on bacteria (Figure 3D). Other CLIP C family members, such as the *Drosophila* proteins PERSEPHONE and GRASS, positively regulate two distinct TOLL pathway serine protease cascades [59].Thus, it is possible that *CLIPC2* is involved in an innate immune cascade in *A. gambiae*. Phylogenetically, *SRPN7* lies within a mosquito-specific expansion cluster and does not cluster with the serpins that have been shown to regulate melanization [35], and its potential role in innate immunity therefore remains elusive. Overall, our data suggest that *CLIPC2* and *SRPN7* are being induced by *P. falciparum* infection through a bacteria-independent mechanism.

### *SRPN7* influences mosquito susceptibility to *Plasmodium falciparum* infection

**Figure 4:**
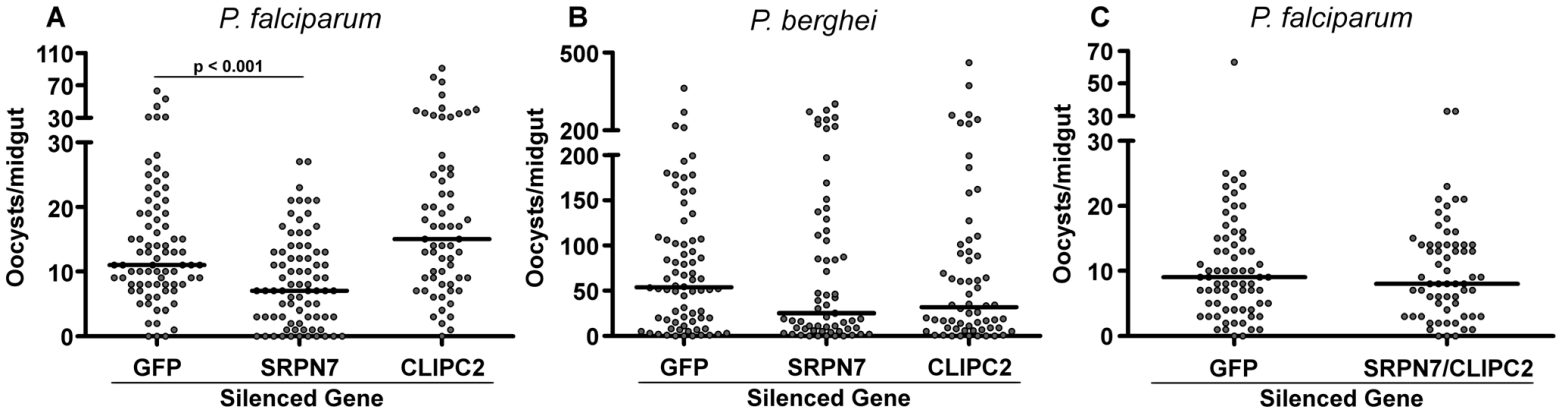
*Plasmodium* infection intensity in aseptic mosquitoes after depleting *SRPN7* or *CLIPC2* through RNAi gene silencing. (A) *P. falciparum* infection intensity following RNAi-mediated depletion of *SRPN7* (Dunn's post test, p<0.05) and *CLIPC2* (Dunn's post test, p>0.05). (B) *P. falciparum* infection intensity following double RNAi-mediated depletion of *SRPN7* and *CLIPC2* (p = 0.87). (C) *P. berghei* infection intensity following RNAi-mediated depletion of *SRPN7* and *CLIPC2* (Kruskal-Wallis test p = 0.42). Circles represent the number of oocysts from a single midgut; horizontal black bars represent the median oocysts in each RNAi treatment. Three independent biological replicates were pooled, and significance was determined by a Kruskal-Wallis test followed by Dunn's post-test in the case of multiple comparisons. Statistical analysis of the double RNAi knockdown was performed using a Mann-Whitney test. RNAi treatments were compared to *dsGFP*-injected control mosquitoes.

We have shown that *SRPN7* and *CLIPC2* transcript abundance changes upon *P. falciparum* infection in a bacteria-independent fashion. To investigate whether *SRPN7* or *CLIPC2* could modulate mosquito permissiveness to *Plasmodium* infection, we used RNAi (RNA interference)-mediated gene silencing in conjunction with *Plasmodium* infection assays under aseptic conditions. Depletion of *SRPN7* resulted in a significant decrease (P<0.001) in the intensity of *P. falciparum* infection in comparison to *GFP* dsRNA-injected controls(Figure 4A). However, there was no significant difference in the prevalence of *P. falciparum* infection between *SRPN7*-depleted mosquitoes when compared to the *GFS* dsRNA-injected controls (Table S3).

Dipteran serpins function in diverse processes ranging from inhibition of signaling cascades, such as the TOLL pathway and the prophenoloxidase activation system, to developmental processes such as morphogenesis [59]–[61]. An earlier study has shown that the *Anopheles SRPN6* has an anti-*Plasmodium* function that is dependent on the mosquito strain/species [34]. The significant reduction in *P. falciparum* infection intensity that we observed upon *SRPN7* depletion suggests that this serpin functions as an inhibitor of an anti-*Plasmodium* defense that involves a serine protease cascade. The fact that, phylogenetically, *SRPN7* does not cluster with the serpins known to be involved in melanization cascades, and the knowledge that the Keele strain mosquitoes used in our study have a weak melanization response and do not melanize *P. falciparum* together suggests that *SRPN7* may be regulating a previously undescribed anti-*Plasmodium* mechanism. Alternatively, the role of *SRPN7* in the Keele strain melanization response could be involved in parasite clearance as opposed to direct melanization [34].

Although *CLIPC2* was upregulated nearly 5-fold in response to *P. falciparum* infection in aseptic midguts, RNAi-mediated depletion of its transcript resulted in no statistical difference in the intensity of *P. falciparum* infection, although there was a slight increase in the overall infection intensity (Table S3). This result may suggest a predominant role for *CLIPC2* in some non-defense-related process that occurs during *Plasmodium* infection, such as tissue repair or the stress response. Alternatively, an anti-*Plasmodium* defense mediated by *CLIPC2* might regulate a single component within a plethora of defenses normally elicited by the endogenous microflora, which we have previously shown can have a significant effect on the intensity of *Plasmodium* infection [11].

We and others have previously shown that different mosquito immune responses are involved in the defense against infection with the two malaria parasite species *P. falciparum* and *P. berghei*. The IMD pathway has been associated with defense against *P. falciparum,* whereas the TOLL pathway is associated with defense against *P. berghei* [4]. We have also shown that *SRPN7* and *CLIPC2* transcripts are induced in aseptic mosquito midguts upon infection with *P. falciparum* but not *P. berghei*. To investigate whether *SRPN7* and *CLIPC2* are regulating a general anti-*Plasmodium* defense or alternatively *Plasmodium*-species-specific defenses, we performed RNAi-mediated gene silencing upon infection with *P. berghei*. Interestingly, independent depletion of either *SRPN7* or *CLIPC2* resulted in no statistical difference in the intensity of *P. berghei* infection when compared to control *GFP* dsRNA-injected control mosquitoes (Figure 4B, Table S3). This result supports the disparity between the mosquito immune response to either *P. falciparum* or *P. berghei* infection and underscores the importance of utilizing the human malaria parasites in mosquito infection studies in order for the results to be of relevance to human disease transmission.

### SRPN7 and CLIPC2 may function in the same serine protease cascade

Since serpins and Clip-domain serine proteases function together as signal transducers and inhibitors in proteolytic signaling cascades, we performed a double knockdown assay of *SRPN7* and *CLIPC2* in aseptic *P. falciparum*-infected mosquitoes to provide a baseline indication as to whether these factors could be functioning in the same cascade, and thereby reciprocally influence their knockdown infection phenotypes. Interestingly, co-silencing of the two genes abolished the effects on *P. falciparum* infection that was observed when each gene was silenced independently (Figure 4C, Table S3). Although the potential for a direct interaction between a serpin and serine protease should be examined by a rigorous biochemical analysis, this experiment, taken together with the bacteria-independent opposite effects of *SRPN7* and *CLIPC2* depletion on susceptibility to *P. falciparum* infection, suggest that *SRPN7* and *CLIPC2* may be operating in the same cascade that regulates anti-*Plasmodium* defense. Alternatively, *SRPN7* and *CLIPC2* could be negative and positive regulators, respectively, of separate processes and thus the result could merely be explained by a canceling effect of silencing both transcripts. Without a biochemical analysis addressing interaction between the two proteins, it may be more accurate to assume that these genes are negative and positive regulators, possibly of the same cascade or independent cascades.

### *CLIPC2* and *SRPN7* influence systemic bacterial infection and the midgut microbiota

**Figure 5:**
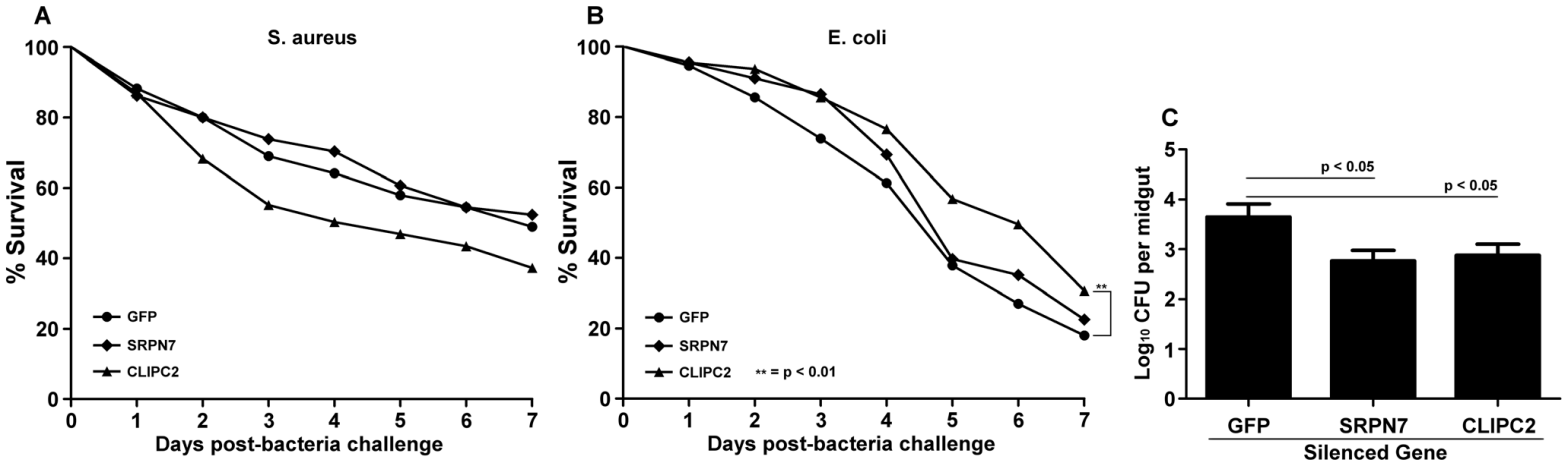
Influence of *SRPN7* and *CLIPC2* silencing on mosquito resistance to bacterial challenge and midgut microbiota proliferation. Adult female mosquitoes were subjected to RNAi-mediated depletion of *SRPN7* or *CLIPC2* transcripts and then challenged with (A) either Gram-positive *Staphylococcus aureus* or (B) Gram-negative *Escherichia coli* bacteria. Depletion of *SRPN7* (p = 0.56) or *CLIPC2* (p = 0.028) had no effect on the survival of mosquitoes challenged with (A) *S. aureus*, whereas there was a significant increase (p<0.01) in the survival of *CLIPC2*-depleted mosquitoes challenged with (B) *E. coli* but not *SRPN7*-depleted mosquitoes (p = 0.18). For both (A) and (B), data were pooled from three independent biological replicates (for A, n = 145; for B, n = 111), and a control group injected with *dsGFP* RNA was included in each replicate. Statistical significance was determined using Kaplan-Meier survival analysis with a log-rank test using Bonferonni's correction for multiple comparisons (significance  = p<0.025. (C) RNAi-mediated gene silencing of *SRPN7* or *CLIP2* resulted in a significant decrease (p<0.05) in the colony forming units (CFU) of cultivable midgut bacteria when compared to *dsGFP*-injected control mosquito midguts. Data were pooled from three independent biological replicates (n = 27 for each dsRNA group), and statistical significance was determined by one-way ANOVA followed by Dunnett's multiple comparison test. Error bars represent the standard error of the mean.

We have previously shown that anti-*Plasmodium* factors also play versatile functions in antibacterial defense and wanted to investigate whether *SRPN7* or *CLIPC2* could play a role in the mosquito's ability to fight systemic bacterial infection, or in the control of its midgut microbiota. While RNAi-mediated depletion of *SRPN7* or *CLIPC2* did not affect the mosquito's survival upon experimental infection with *S. aureus*, mosquitoes depleted of *CLIPC2* showed increased survival when infected with *E. coli*, suggesting that *CLIPC2* may be a host factor for this bacterium (Figure 5A, B). The mosquito's midgut microbiota needs to be under continuous immune control to avoid an over-proliferation that could be detrimental to the insect. We have previously shown that factors of the IMD immune pathway play a crucial role in controlling the midgut microbiota, and conversely, that the microbiota is responsible for priming basal immune activity [10], [11]. Surprisingly, independent silencing of *SRPN7* and *CLIPC2* resulted in a significant decrease of the mosquito's midgut microbiota, suggesting that these putative immune factors act as agonists of the mosquito's natural midgut bacteria, through an unknown mechanism (Figure 5C). The midgut microbiota are predominately Gram-negative [62], and the decrease in the midgut bacteria in response to the silencing of *CLIPC2* corroborates the increase in survival that was observed in *E. coli*-challenged mosquitoes depleted of *CLIPC2*. Whereas silencing of *SRPN7* resulted in a decrease in the mosquito's midgut microbiota, silencing of this gene had no effect on survival after systemic bacterial challenge, suggesting that the function of *SRPN7* may may be associated with the midgut as opposed to the fat body. Although we have hypothesized that *SRPN7* and *CLIPC2* operate in the same or different serine protease cascades to activate defense mechanisms, the effects of *SRPN7* and *CLIPC2* depletion on resistance to bacterial infection and the microbiota, respectively, cannot be fully explained by this model and suggests that these two factors may be associated with multiple and functionally diverse serine protease cascades. Nevertheless, our results further corroborate the functional unrelatedness of *SRPN7* and *CLIPC2* with the bacteria-dependent IMD pathway-mediated anti-*Plasmodium* defense system and highlight the functional versatility and complexity of mosquito immune factors.

### *SRPN7* and *CLIPC2* do not regulate the IMD immune pathway

**Figure 6:**
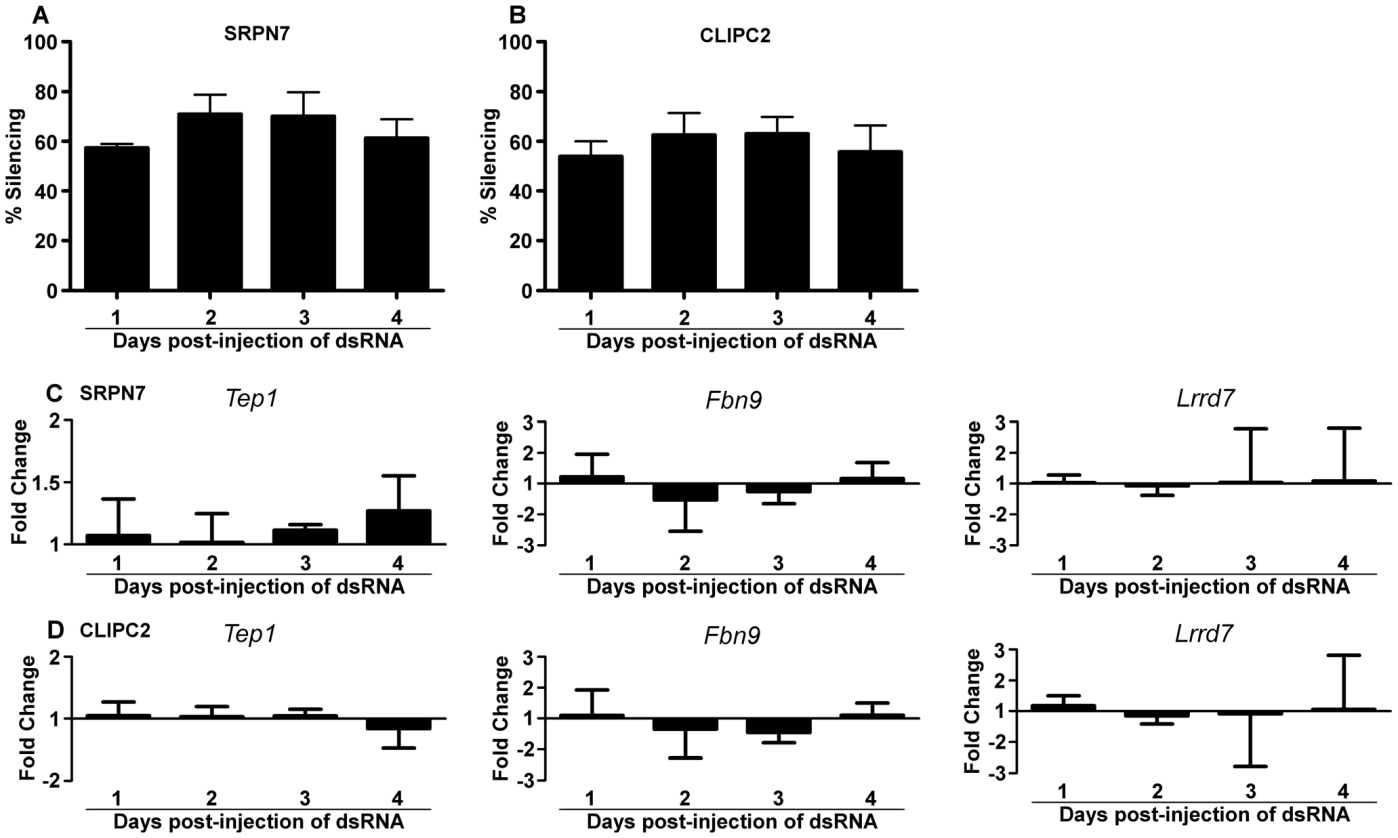
*SRPN7* or *CLIPC2* depletion has no effect on the expression of IMD pathway-regulated anti-*P. falciparum* genes. (**A**) Silencing of *SRPN7* and *CLIPC2* was measured over a period of 4 days by qRT-PCR. Fifteen midguts, from aseptic mosquitoes, were pooled on each day post-injection, and the results represent the mean silencing for two independent biological replicates. Error bars represent the standard error of the mean. Expression of *TEP1, FBN9,* and *LRRD7* genes following single knockdown of (C) *SRPN7* or (D) *CLIPC2*. Bars represent the -fold change in expression of the listed genes on days 1–4 post-dsRNA injection, as compared to *dsGFP*-injected controls. qRT-PCR was used to assess changes in expression of the genes indicated above each graph. Error bars represent the standard error of the mean for three biological replicates Statistical analysis was performed at each time point by one-way analysis of variance (ANOVA) followed by Dunnett's post-test to account for multiple comparisons; all genes showed no significant difference in expression when compared to *dsGFP*-injected controls (not depicted).

Although serpins and clip serine proteases have been identified as regulators of the TOLL pathway, it is unclear whether similar cascades are involved in regulating the activation of the IMD pathway. The TOLL pathway is primarily responsible for immunity against the rodent parasite *P. berghei,* whereas the IMD pathway is associated with immunity against the human parasite *P. falciparum* [4]. Since *SRPN7,* and to some degree *CLIPC2,* appear to modulate *P. falciparum* infection intensity, we hypothesized that these genes could be involved in regulating the IMD pathway. In order to determine whether *SRPN7* or *CLIPC2* is involved in activating the IMD pathway, we used qRT-PCR to measure the abundance of three IMD-pathway-regulated, anti-*Plasmodium* gene transcripts (*TEP1, FBN9, LRRD7*) at 24, 48, 72, and 96 hours following RNAi-mediated gene silencing of *SRPN7* or *CLIPC2* in aseptic mosquitoes (Table S1). We chose to monitor the expression of these genes over 4 days because of the previously reported temporal regulation of IMD pathway-driven gene transcript abundance [63]. The silencing efficiency over 4 days averaged 62% for *SRPN7* and 59% for *CLIPC2*, respectively (Figure 6A, B). Depletion of either *SRPN7* or *CLIPC2* did not influence the transcript abundance of *TEP1, FBN9,* or *LRRD7* over the course of the experiment when compared to *GFP* dsRNA-injected control mosquitoes (Figure 6C, D). These three potent anti-*Plasmodium* genes were previously found to be upregulated by the IMD pathway upon silencing of the negative regulator *CASPAR* and upon overexpression of the IMD pathway transcription factor *REL2* [6]. In our earlier studies we have also shown that the abundance of *SRPN7* and *CLIPC2* transcripts is not regulated by the IMD pathway [6]. *FBN9* is induced by the native microbiota in the mosquito midgut, and all three genes are involved in controlling its microbiota as well as in systemic bacterial challenge [5], [11]. Furthermore, none of these genes were upregulated in the *P. falciparum*-infected aseptic midguts. In the absence of bacteria, *SRPN7* and *CLIPC2* were upregulated in the *P. falciparum*-infected aseptic midguts, and depletion of *SRPN7,* and to some degree *CLIPC2,* modulated the intensity of *P. falciparum* infection, yet these genes do not appear to regulate the expression of anti-*Plasmodium* factors through the IMD pathway. These findings suggest that bacteria- and IMD pathway-independent anti-*P. falciparum* defenses exist, and they underscore the complexity of the mosquito's anti-*Plasmodium* immune mechanisms.

## Conclusions

The *Anopheles* mosquito's anti-*Plasmodium* defense system is actively engaged in limiting parasite infection of the midgut epithelium by mounting immune responses against the ookinetes in the midgut lumen and epithelium [8]. While these immune responses have been shown to be regulated to some extent by midgut microbiota-mediated activation of the IMD pathway, we show here for the first time that other, as yet uncharacterized, microbiota- and IMD pathway-independent immune responses also participate in limiting *P. falciparum* infection. The potential affiliation of *SRPN7* and *CLIPC2* with a serine protease activation cascade suggests that these genes are controlling the activation of an effect mechanism, rather than representing effectors themselves. The regulation and parasite killing mechanism of these defenses appear to be quite different from those previously characterized since (a) *SRPN7* and *CLIPC2* are not regulated by, nor do they regulate, the IMD pathway and (b) they act against *Plasmodium* independently of the midgut microbiota. The observation that *SRPN7* and *CLIPC2* were only regulated in the *P. falciparum*-infected aseptic midguts, strongly suggests that an upstream pattern recognition molecule is sensing *P. falciparum* and culminating in the activation of an undescribed pathway. Alternatively, a molecule upstream of SRPN7 and CLIPC2 could be sensing damage to the midgut epithelium mediated by *P. falciparum* invasion. *SRPN7* and *CLIPC2* were neither induced by nor involved in anti-*P. berghei* defense, suggesting an association with defense against *P. falciparum* and demonstrating the ability of the mosquito immune system to discriminate between infections of closely related pathogens. A *P. falciparum*-specific defense pathway could be exploited in a translational approach to control *Plasmodium* in the mosquito, as opposed to the human host. A biochemical analysis of their interacting partners will be necessary to confirm that these molecules are true partners and that they regulate the same effector mechanism. In summary, we have discovered *SRPN7* and *CLIPC2* in the bacteria-independent, *Plasmodium* infection-responsive transcriptome and demonstrated the existence of IMD pathway-independent defenses against *P. falciparum*.

## Supporting Information

Table S1 Primers used in this study. PCR primer sequences used in this study. “Gene Target” displays the target gene of the corresponding primer. “AGAPID” lists the Vectorbase identifier if applicable such as in the case of a primer designed to produce double-stranded RNA for targeted gene silencing. “Primer name” is an arbitrary identifier for a particular primer sequence. “Primer sequence” displays the forward and reverse primers designed for a specific gene. “Primer use” shows the use of a given primer (i.e. RNAi is for generating double stranded RNA whereas qRT-PCR indicates a primer was used in real-time PCR analyses).
(XLS)

Table S2 Microarray data analyzed in this study. Master tab: Microarray assayed Log2 transformed transcript abundance ratio of corresponding genes using a cutoff of 0.75 for upregulation and −0.75 for downregulation. The “Gene Description” column provides a gene name for the corresponding transcript if one is available. The “Functional Group" indicates the assignment of a transcript to a particular functional group dependent on the predicted function of the gene (I/A: putative immunity and apoptosis; R/S/M: oxidoreductive, stress-related and mitochondrial; C/S: cytoskeletal, structural; MET: metabolism; R/T/T: replication, transcription, translation; P/D: proteolysis, digestion; TRP: transport; DIV: diverse; UKN: unknown functions.). The “AGAP-ID” column lists the Vectorbase identifier for a particular gene (http://www.vectorbase.org). The following four columns display Log2 ratio values from either the midgut or carcass between the aseptic and septic treatments.
(XLSX)

Table S3 Supplementary data from Figure 4. Supplementary data from *Plasmodium* infection assays displayed in Figure 4. Includes number of samples assayed, mean, median, % change, and prevalence of oocysts.
(XLS)
